# Proteomic analysis of prolactinoma cells by immuno-laser capture microdissection combined with online two-dimensional nano-scale liquid chromatography/mass spectrometry

**DOI:** 10.1186/1477-5956-8-2

**Published:** 2010-01-29

**Authors:** Yingchao Liu, Jinsong Wu, Guoquan Yan, Ruiping Hou, Dongxiao Zhuang, Luping Chen, Qi Pang, Jianhong Zhu

**Affiliations:** 1 Department of Neurosurgery, Shandong Provincial hospital affiliated to Shandong University, Jinan, 250021, China; 2 Shanghai Neurosurgical Center, Department of Neurosurgery, Huashan Hospital, Shanghai Medical College, Fudan University, Shanghai, 200040, China; 3 Department of Chemistry, Fudan University, Institutes for Biomedical Sciences, Fudan University, Shanghai, 200433, China; 4 Department of Gastroenterology, Shandong Provincial Qianfoshan Hospital affiliated to Shandong University, Jinan, 250014, China; 5 National Key Lab for Medical Neurobiology, Institutes of Brain Sciences, Fudan University, Shanghai, 200032, China

## Abstract

**Background:**

Pituitary adenomas, the third most common intracranial tumor, comprise nearly 16.7% of intracranial neoplasm and 25%-44% of pituitary adenomas are prolactinomas. Prolactinoma represents a complex heterogeneous mixture of cells including prolactin (PRL), endothelial cells, fibroblasts, and other stromal cells, making it difficult to dissect the molecular and cellular mechanisms of prolactin cells in pituitary tumorigenesis through high-throughout-omics analysis. Our newly developed immuno-laser capture microdissection (LCM) method would permit rapid and reliable procurement of prolactin cells from this heterogeneous tissue. Thus, prolactin cell specific molecular events involved in pituitary tumorigenesis and cell signaling can be approached by proteomic analysis.

**Results:**

Proteins from immuno-LCM captured prolactin cells were digested; resulting peptides were separated by two dimensional-nanoscale liquid chromatography (2D-nanoLC/MS) and characterized by tandem mass spectrometry. All MS/MS spectrums were analyzed by SEQUEST against the human International Protein Index database and a specific prolactinoma proteome consisting of 2243 proteins was identified. This collection of identified proteins by far represents the largest and the most comprehensive database of proteome for prolactinoma. Category analysis of the proteome revealed a widely unbiased access to various proteins with diverse functional characteristics.

**Conclusions:**

This manuscript described a more comprehensive proteomic profile of prolactinomas compared to other previous published reports. Thanks to the application of immuno-LCM combined with online two-dimensional nano-scale liquid chromatography here permitted identification of more proteins and, to our best knowledge, generated the largest prolactinoma proteome. This enlarged proteome would contribute significantly to further understanding of prolactinoma tumorigenesis which is crucial to the management of prolactinomas.

## Introduction

Prolactinomas are the most common pituitary tumors, representing 25%-44% of all pituitary adenoma cases [1]. Although most are pathologically benign and grow slowly, prolactinomas show many symptoms in patients: amenorrhea, galactorrhea and dysgenesis in female patients and infertility and erectile dysfunction in male. Moreover, a number of prolactinomas belie their histology by perisellar invasion and postoperative recurrence. Comprehensive molecular dissection of prolactinoma pathogenesis is demanded for further understanding of this kind of tumors. Increasing evidences suggest that characterization at DNA or RNA level alone would not be sufficient to elucidate the mechanisms of this disease as lots of posttranslational modifications exist and pituitary adenoma proteomics would offer an efficient means for a comprehensive analysis of prolactinoma. Desiderio's research on human pituitary adenoma proteome [2-4], plus newly developed proteomics methodologies paved a path for further proteomics studies of prolactinomas.

LCM allows the isolation of even single cell and immunohistochemistry (IHC) staining could help to distinguish specific cell populations, thus, employment of immuno-LCM could pick up certain cell populations with specific immuno-phenotype from complex tissues according to their antigen expression to ameliorate the problem of tissue heterogeneity in proteomic research [5,6].

Two-dimensional gel electrophoresis (2-DE) separates mixture of proteins by their isoelectric point (pI) and molecular weight (MW), producing a high-resolution protein map from which protein spots can be processed individually [7]. While 2-DE serves as a powerful separation tool, shotgun proteomics combining LC-MS/MS have emerged as a technique of choice for large-scale protein studies due to its superior throughput and sensitivity [8].

In a typical shotgun proteomics experiment, a complex protein sample is digested into peptides followed by separation by 2D-nanoLC/MS, resulting peptides were loaded into a mass spectrometer for fragmentation and sequencing. Sequencing results were used to identify the parent proteins via database searching. High performance duty cycle instruments can fragment hundreds of peptides in a single LC-MS/MS experiment. However, a prolactinoma, even from microdissected sample, contains thousands of tryptic peptides. At a given LC elution time, multiple coeluting peptides would quickly overwhelm other MS/MS acquisition speed of duty cycle instruments. In order to identify proteins from a complex mixture with a dynamic range of at least 10^5 ^[9], it is crucial to develop technologies with extremely good resolving power and extraordinary sensitivity. It is obvious that these challenging tasks will not be achieved with a single analytical technique but with a combination of different separation and detection techniques.

As shotgun proteomics could generate such a large number of protein identifications via high-throughput 2D separation, we combined a 2D peptide-level separation performed by coupling strong cation exchange (SCX) with reverse phase (RP) plus SDS-PAGE protein prefraction to increase dynamic range and proteome coverage for bottom-up shotgun proteomics [10,11].

In our recently published study we have reported a novel method for immuno-LCM in frozen sections of prolactinomas [12]. Microdissected prolactin cells were solubilized followed by protein separation on SDS-PAGE gel. The gel slices were then digested and fractions of the resulting peptides were separated by 2D-nanoLC/MS and characterized by tandem mass spectrometry [13]. All MS/MS spectrums were searched by SEQUEST against the human International Protein Index (IPI human, version 3.45 fasta with 71983 entries) database to provide a profile of prolactinoma proteome. A proteome consisting of 2243 proteins was thereby acquired. Compared with previous pituitary adenoma proteome research [2], our proteome contains more than twenty times the number of proteins identified by an initial 2-DE map of extracts from a pituitary adenoma. Categories of the proteome include low-abundance proteins, membrane proteins, cyclin dependent kinases (CDK) and phosphoproteins.

## Results

### Identification and characterization of prolactinoma proteome

**Figure 1:**
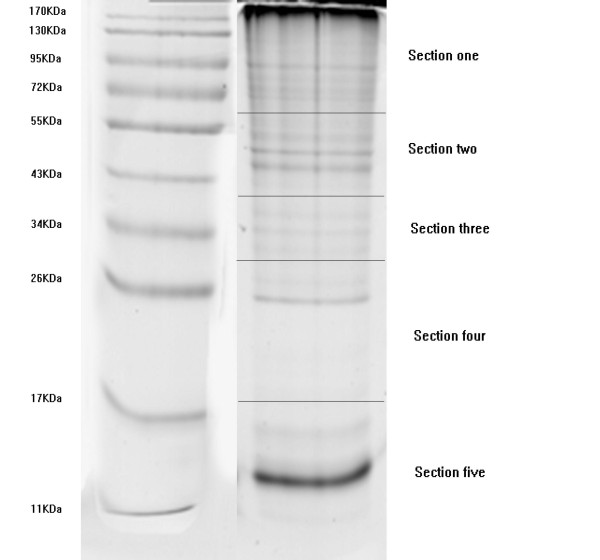
**SDS-PAGE of PRL cells**. PRL cells collected from LCM were lyzed with urea (7 M) and thiourea (2 M). Solubilized proteins were separated via SDS-PAGE and stained with Coomassie blue. The gel was cut into five sections based on molecular mass and each section was subjected to in-gel digestion with trypsin.

Since one shortcoming of LCM is the number of pure cells for proteomic analysis was limited, only 100,000 cells were used in this work. Thus, separation steps were accordingly limited to reduce the sample loss. Proteins from this small number of cells were fractionated by SDS-PAGE first and the resolved protein gels were then cut into five sections according to several distinct protein bands observed within this gel (Fig. 1). The five sections cut from the SDS-PAGE gave the optimal results in terms of the detection sensitivity and the number of proteins identified.

Although some proteins were identified from more than one gel section, each of the five gel sections provided a large number of unique protein identifications. In this process, the peptide fragmentation pattern was matched to the peptides constructed from the theoretical human International Protein Index (IPI) database. A less than 5% false positive rate was estimated through the reverse database search (reverse the protein sequences in the human protein database). Such stringent criteria in our experiment would eliminate most peptides that would falsely increase the number of identified proteins (data not shown).

**Figure 2:**
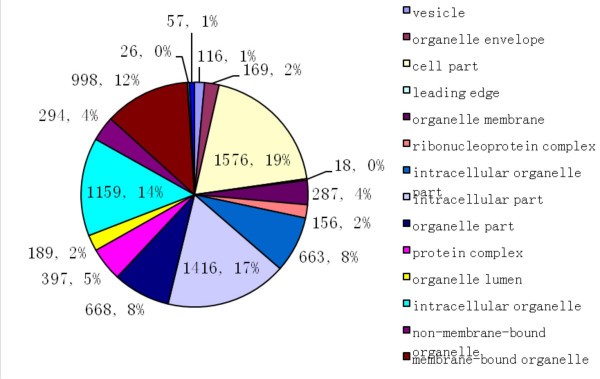
**Categories of identified proteins in prolactinomas by cell components**. The distribution of enrolled proteins in DAVID 2009 was 16% of cell part, 15% of intracellular part, 15% of intracellular, 12% of vesicle, 4% of protein complex and other 11 fractions according to cell components.

The matched peptide sequence analyzed by 2D-nanoLC/MS was identified based on the fragmentation pattern (MS/MS) as well as mass accuracy of peptide molecular weight (within 10 ppm). From the digested peptides of the five gel slices, 122807 MS/MS spectrums were acquired and these data were used for SEQUEST-based database searches. The results from the five gel slices were combined as shown in Table 1; 28784 peptides including 115 for 5^+ ^ions, 1373 for 4^+ ^ions, 5972 for 3^+ ^ions and 21328 for 2^+ ^ions were submitted, and after removal of redundant proteins, 2249 proteins were finally identified. To our best knowledge, after eliminating six keratin proteins from skin or hair [2], the 2243 identified proteins represent the largest database of human pituitary to date(Additional file 1). Among these identified 2243 proteins, 1802 proteins were identified with at least two unique peptides representing identifications of high probability. Cellular distribution of these identified proteins (as a percentage) was analyzed using a bioinformatic analysis tool-The Database for Annotation, Visualization and Integrated by Discovery [14] (DAVID 2009, Version 6) in Fig. 2.

**Table 1:** Number of proteins identified in each gel section

Section number	Section 1	Section 2	Section 3	Section 4	Section 5
Number of protein	747	805	769	672	346
Protein matched ≥ 2 peptides	608	662	647	546	265

## Discussion

### Prolactinoma proteome by immuno-laser capture microdissection

The global analysis of prolactinoma protein expression complements genomics analysis and may provide further insight into post-translational modifications affecting cellular function. The pituitary adenoma represents a complex heterogeneous mixture of cells including prolactin (PRL), endothelial cells, fibroblasts, and other stromal cells. Since Emmert-Buck MR *et al *[15] introduced LCM in 1996, this technique has been widely used to isolate specific cell types from mixture tissues for biomedical research especially for proteomic analysis [16-19]. Although this proteomic technique has been widely used with regard to different aspects of neurological diseases [20], there are few data available about proteomic analysis of human pituitary adenoma [2,21-24]. Therefore, in two earlier reviews about pituitary adenoma research Lloyd RV and Xianquan Zhan pointed out that LCM is a proposed and perspective method for pituitary adenoma proteomic analysis [16,25]. One research about normal pituitary by immuno-LCM had proved it a valuable technique for RNA analysis [26]. Our group has recently demonstrated a novel method to acquire human pure prolactin cells for proteome analysis by immuno-LCM [12]. In this study we used SDS-PAGE combined with 2D-nanoLC-MS/MS strategies for proteome study to identify a total of 2243 unique proteins in prolactin cells. To our knowledge, this is the most comprehensive proteomic analysis of human pituitary adenoma to date.

Our previous experiments showed that TritonX-100 concentration for immunostaining was optimal at 0.2% for 4 min by extracting the lipid proteins from the adenoma cell surface [12]. Since hematoxylin [27] and IHC [28] stained tissues have been successfully employed with mass spectrometry, in order to test the compatibility of the pretreatment of 0.2% TritionX-100 solution with mass spectrometry, protein recovery from prolactinoma sections without treatment was directly compared with that from sections processed with the pretreatment of TrtionX-100, and no significant differences in protein recovery were observed between the two protocols (data not shown). The results demonstrated that the pretreament of 0.2% TrtionX-100 for 4 min did not affect proteomic analysis. In addition, the novel pretreatment provided improved morphological discrimination of prolactin cells for successful and easy LCM.

### A comparison between two pituitary adenoma proteomes

In an earlier pituitary adenoma proteome research by 2-DE [2], the authors identified 111 proteins. Most of the 2-DE-separated spots were distributed in the area of pH 4-8 and mass 20-80 kDa. The mean MW of the proteome was 41.8 kDa, and the highest MW protein was "complement C3 precursor (fragment)" which had a 187.1 kDa MW and the lowest MW protein was "Myosin light chain alkali. Smooth muscle isoform" with a 6.5 kDa MW. The mean pI was 6.13, and the highest pI protein was "RAB GDP dissociation inhibitor beta" which had a 9.8 pI and the lowest pI protein was "Myosin light chain alkali" with a 4.38 pI respectively. For the 39 LC-ESI MS/MS characterized proteins, the number of matched peptides ranged from 2 to 18. The sequence coverage of these MS/MS data ranged from 7% to 56%. While for the 96 MALDI characterized proteins, the number of matched peptides ranged from 4 to 19. The sequence coverage of these PMF data ranged from 7%-73%.

Most of the identified proteins (70%) MW in our research theoretically distributed between 20 kDa and 100 kDa (mean MW = 75.3 kDa). Nevertheless, there were still some proteins with an extremely low or high MW in our database. Thirteen proteins with MW <10 kDa were identified; the smallest protein is the "up-regulated skeletal muscle growth protein 5" (IPI 00063903.5) which has a theoretical MW of 6457 Da (identified from gel section 5). A total of 143 proteins with MW of >200 kDa were identified; the largest protein identified in this work was "isoform of titin" (identified from protein section 1) which has a theoretical MW of 3805759 (IPI 00023283.3). The pI range of the proteome was between 3.93 and 12.05 (mean pI = 6.94), among which 16.7% of proteins were high basic proteins with pI greater than 9. The lowest and the highest pI protein were "isoform 1 of acidic leucine-rich nuclear phosphoprotein 32 family member B" (IPI 00007423.1) in section 2 (pI = 3.93) and "isoform 1 of serine/arginine repetive matrix protein 2 (IPI 00782992.3) in section 4 (pI = 12.05). Those proteins either with extreme molecular weights or pIs can be rarely detected by traditional 2-DE analysis of pituitary adenoma. In this new pituitary adenoma proteome, the number of matched peptides ranged from 1 to 128. The sequence coverage of these MS/MS data ranged from 0.1%-87%. (Table 2). The smallest sequence coverage in our research was quite lower than that of Zhan's results. There are many more high MW proteins identified in our report with only two or more peptides were matched to these proteins. However, low sequence coverage these proteins have, these proteins were identified with a high probability.

**Table 2:** Quality comparison of pituitary proteome by 2-DE and 2D-nanoLC/MS Table 2 indicated the quality comparison between the two pituitary adenoma proteomes on the number of matched peptides, matched peptides ratios, the percentage sequence coverage, pI range, MW range, mean pI and mean MW.

Methods	Number	Matched peptides	Sequence coverage	Mean pI	pI range	Mean MW (KDa)	MW range
MS/MS	39	2-18	7-56%	6.13	4.4-9.4	41.8	12.1-180.7
PMF	96	4-19	7-73%				
MS/MS	2249	1-128	0.1-87%	6.95	3.9-12.1	75.3	6.5-380.6

**Figure 3:**
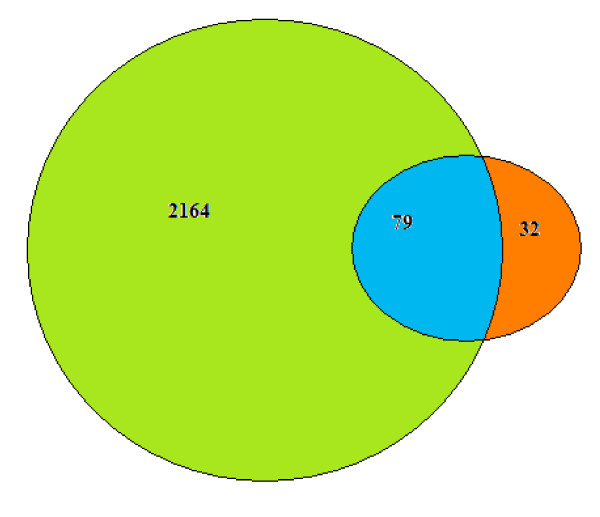
**Protein overlaps of two proteomes**. Total proteins are indicated for each proteome with two circles; subsets in common (in blue) are indicated within the diagram. It indicates that most proteins identified in 2-DE (in brown) are overlapped with what extracted from the prolactinomas by immuno-LCM (in green).

Using SDS-LC-MS/MS, Tomazella *et al *[29] identified 251 total cellular proteins from resting human neutrophils, that is more than ten times of proteins identified by an initial proteome analysis of human neutrophils and almost five times of proteins identified by the first 2-DE map of extracts of rat polymorphonuclear leukocytes. With SDS-2D-nanoLC-MS/MS in this research, the total number of identified proteins was substantially increased to 2243 and there are 79 in common between the two proteomes (Fig. 3). This might be explained with: (i) any proteome carried out by the present proteomic technology is only part of the "whole" proteome. There were differences in any different proteomes even they were from the same sample and MS; (ii) trans-membrane proteins and Less abundant proteins were not easily extracted or identified in 2-DE studies either due to their hydrophobicity and aggregation during IEF, or low abundance [30]; (iii) In fact, many of the spots detected in the gels were actually variations of the products of post-translationally modified proteins [31] that were not usually detected by shotgun sequencing and thus can be a limiting factor of the shotgun approach; (iv) Pituitary adenomas were divided into six types according to the hormone secreted (GH,PRL,ACTH,TSH,LH,FSH) and each type of adenoma has its unique protein expression characters. Our proteome was from the pure prolactin cells of prolactinomas by immuno-LCM while the other proteome by Xianquan Zhan was from the whole non-functional adenoma tissues [2], which may be the main reason for the difference.

In our experience, 2-DE identified and revealed relative distribution of more abundant proteins whereas LC-ESI-MS/MS identified less abundant proteins and hydrophobic proteins not amenable to detection by 2-DE. After the immuno-LCM procedure, cells were lyzed and fractionated by molecular weight differences using SDS-PAGE, because SDS-PAGE was not only an effective pre-fractionation method [32], but also provided approximate molecular weight information for identified proteins [33]. One previously described immunostaining method for proteomics was based on immunogold detection and involved very short incubation times (5 min) and very high antibody titers (1/25-1/5) [34]. Such extreme ICH conditions have several potential drawbacks. In many cases, especially when using polyclonal antibodies, the background staining could be very high. In contrast to previously reported studies, we have demonstrated the membrane-mounted sections pretreatment of the frozen prolatinoma sections with 0.2% Triton X-100 with a less drastic modifications to conventional IHC staining protocols was highly compatible with 2D-LC shot gun proteomic studies. The results obtained from this research clearly proved the capability of 2D-nano LC-MS/MS tandem mass spectrometry for the separation and identification of complex peptide samples compared with traditional 2-DE methods. Although some differences existed, both proteomes complemented each other and contributed to the elucidation of the mechanisms of prolactinoma.

### Bioinformatics of the prolactinoma proteome

**Figure 4:**
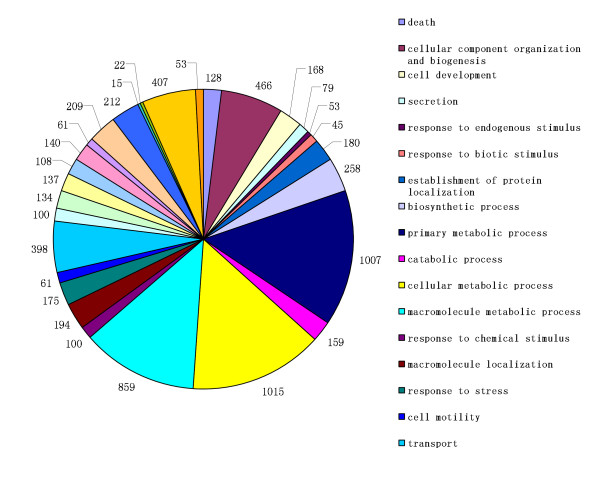
**Categories of identified proteins in prolactinomas by biological process**. Proteins in the proteome participate in pituitary hormones, cellular signals, biosynthetic process, cellular metabolic process, cell development, response to endogenous stimulus, transportation, *etc*.

The protein biological process and functional categories provided an overview of the human pituitary adenoma tissue proteome based on the known or postulated functions of the identified protein. Analysis of the proteome with DAVID revealed a notable identification of proteins participating in pituitary hormones, cellular signals, biosynthetic process, cellular metabolic process, cell development, response to endogenous stimulus, transportation, *etc*. Such functional groups are in agreement with pituitary biology in previous research [35-38]. The full classification of the proteins according to their biological functions is demonstrated in the pie chart in Fig. 4. Among the identified proteome, 2164 proteins that are potentially relevant to the biological process of proteome not yet been described in any of the papers researching pituitary adenomas cited above. Many proteins play complex biological roles, so in our research the total number of all subgroups was larger than that of the whole proteome. Our data suggests that SDS-PAGE protein prefraction coupled with 2D-nanoLC/MS peptide separations was still required to maximize the separation capacity to meet the challenge of the complexity of the prolactinoma proteome.

Several functionally important proteins pertinent to biological action of the prolactinoma were identified in this database which had been widely investigated in different aspects of pituitary adenoma.

Cyclin proteins: Cyclins and cyclin dependent kinases (CDK) (Isoform 1 of CDK Substrate, cyclin G-associated kinase and isoform 1 of calcyclin-binding protein were identified in the proteome database) are essential for cell cycle regulation in eukaryotes. Active cyclin-CDK complexes drive cells through cell cycle phases by phosphorylating the protein substrates that are essential for the transition to the next phase [39]. Several researches have found that prolactinomas significantly express cyclin gene and some cyclin genes may be a marker for invasion of prolactinomas and may also be related to tumor recurrence [40].

G-type cyclins (G1 and G2): G-type cyclins seem to participate in G2-M arrest in response to DNA damage [41]. G-type cyclins bind to GAK that is a serine/threonine cyclin G-associated kinase involved in epidermal growth factor receptor signaling [42]. G-type cyclins are targets of p53 and seem to be involved in the ATM-p53-Mdm2 pathway [43].

The 14-3-3 family of proteins identified in the list had a further significance. The 14-3-3 (Ywha or 3-monooxygenase/tryptophan 5-monooxygenase activation protein) family consists of highly conserved, approximately 30 kDa acidic proteins that form homodimers or heterodimers [44]. In mammals, up to seven homologous isoforms of this kind of proteins have been described (β γ δ ε ζ η θ) [45] (14-3-3 protein θ/δ, 14-3-3 protein ε, isoform long of 14-3-3 protein β/α, 14-3-3 protein η, 14-3-3 protein γ and 14-3-3 protein θ were detected in this research). The 14-3-3 family members mediate signal transduction by binding to phosphoserine-containing proteins. It interacts with CDC^25 ^(cell division cyclin^25^) phosphatases, RAF1 and p^27 ^proteins, suggesting its role in diverse biochemical activities related to signal transduction, such as cell division and regulation. All isoforms of 14-3-3 proteins recognize two high affinity phosphorylation dependent 14-3-3 binding motifs: R(S/X) XpSXP (mode 1) and RXXXpSXP (mode 2) where pS is a phosphoserine. 14-3-3 plays a role in the regulation of various cellular signaling events [46]: (i) change the ability of target protein to interact with other partners; (ii) modify the cellular localization of target protein; (iii) bridge two target proteins; (iv) change the activity of the target protein; and (v) protect their target protein. A recent research has been shown that 14-3-3 is able to act as a sub-cellular localization modifier for several phosphorylated proteins [47].

The p^27 ^protein (IPI 00010860.1) belongs to the large family of cyclin-dependent kinase inhibitors (CDK1). The p^27 ^protein regulates the progression of the cell cycle from the G1 to the S phase. The levels of p^27 ^protein in much malignant neoplasm are lower than those in normal tissues, suggesting that p^27 ^may act as a suppressor and has a prognostic significance [48]. P^27 ^protein has been identified to cause pituitary adenomas [49,50]. The very low levels of p^27^, both native and Thr-phosphorylated, were seen in pituitary carcinoma [51]. In addition, recurrent pituitary adenomas show significantly lower p^27 ^expression levels (47%) than non-recurrent tumors (67.4%) [52]. In one recent research, the molecular analysis of human pituitary neoplasm has corroborated that the cell cycle inhibitor p^27^Kip1 deregulation is significantly implicated in pituitary tumorigenesis. In particular, proteins involved in cyclin-dependent kinase regulation or the pRb pathway are altered in nearly all human pituitary tumors [53].

## Limitations

A pooled sample for proteomic profile analysis would represent a more comprehensive proteome of prolactinomas, but just because the mixture of many samples, the concentration of some protein would be too low to be detected by mass spectrometry and thus the integrity of the proteome was affected. Our team's next research would focus on the label-free comparative proteomic research, each prolactinoma sample will be analyzed by shotgun proteomics to produce mass spectral peak intensities and spectral counts, and the relative intensities of sequence ion fragment peaks within an MS/MS spectrum will be compared by statistics to get the differential proteins, and at the same time, an enlarged prolatinoma proteome will be obtained.

Though low density Triton X-100 had been proved to have no obvious affect on proteomic analysis [54], the pretreatment of TritonX-100 to improve the intensity of positive prolactin cells in LCM will definitely resulted in the loss of some proteins in cell membrane. The paraffin embodied pituitary adenomas sections need no Triton X-100 treatment during the IHC for LCM which might be another potential bank for further pituitary adenoma proteomic research. Our team had finished the experiment to get prolactin cells by LCM from paraffin embodied pituitary adenoma samples and the proteins number was nearly equal to that from frozen samples (data not shown).

## Conclusions

In the present study, the proteome database of prolactinomas demonstrated that immuno-LCM coupled with 2D-nanoLC/MS mass spectrometry, should enable the analysis of protein expression profiles in defined group of prolactin cells, allowing us to extend the present proteome research to a wide range of other cell types of pituitary adenomas. This enlarged proteome will serve as a more comprehensive complement of understanding of previous pituitary adenoma proteome researches and will also provide a basis for label-free comparative proteomics to find the key cellular events of pituitary tumorigenesis in our future researches.

## Materials and methods

### Immuno-laser capture microdissection of prolactinomas

This study was performed under approval of the ethics committee of Huashan Hospital, Fudan University and informed consents were received from each patient. Eleven prolactinomas (5 males and 6 females) were collected during operations and cut approximately 3 mm in diameter for the subsequent frozen sections. Eight-micrometer sections on membrane-mounted slide (Microdissect GmbH, Germany) were collected in the cryostat (Leica CM 1900, Germany) and immediately fixed in 100% ethanol for 15 min; the sections were incubated in 0.2% Triton X-100 with Tris-Buffered Saline (TBS) for 4 min and then incubated at 1:300 dilution of primary anti-rabbit polyclonal prolactin antibody (Dako Cytomation, Copenhagen, Denmark) in the TBS solution for 30 min in a humid chamber, and then were washed twice for 3 min each in TBS. Afterwards, all sections were incubated with biotinylated "Universal" secondary antibody (Dako Cytomation Copenhagen, Denmark) for 20 min at room temperature. Finally, all sections were developed with diaminobenzidine (Dako Copenhagen, Denmark) for 1 to 2 min and counterstained with hematoxylin (Sigma Diagnostics, St. Louis, MO) and the sections were ready for LCM. A Varitas™ Arcturus LCM, combined with IR/UV system (Arcturus Molecular Devices, CA, USA), provide a narrow ultraviolet laser beam to "draw around" and cut out the cells of interest by photo volatilization (20×). A cap would be placed on the tissue after the UV laser cut the desired area. The IR laser would tack the polymer to collect the desired cells. Laser cut parameters were: spot size = 4 μm, power = 80 mW, pulse duration = 15 ms. Time between pulses = 400 ms. After a successful immuno-LCM, thirty 8 μm prolactinoma sections from eleven prolatinomas provided an approximately 6.0 mm^2 ^surface area which was equal to 100,000 prolactin cells for the subsequent shotgun proteomic analysis.

### Cell lysis and SDS-PAGE gel protein separation

Lysis buffer, containing urea (7 M) and thiourea (2 M), was added to a 0.5 ml Eppendorf tube connected to the LCM cap. Captured cells were solubilized by a series of 20 ms sonication bursts, followed by a 20 s ice cooling, which was repeated five times. The entire extract (~40 μl) was loaded on a gel (SDS-PAGE, 4%-12% gradient) to separate proteins by molecular weight (MW) [13]. The MWs of the proteins were estimated by a comparison with the migration of standard proteins. This plot yielded a straight line; the MWs of the unknown proteins were determined by this plot, using the mobility calculated from the migration of the protein spot. After staining with Coomassie blue, the gel was cut into five individual sections with molecular mass ranges. Each section was further minced into small pieces (approximately 0.5 mm^2^) and subjected to destaining cycles of gel dehydration with ACN and rehydration with NH_4_HCO_3 _buffer (0.1 M, pH 8.0) in order to remove the Coomassie stain. This procedure was repeated up to three times until no visible Coomassie stain remained. The in-gel proteins were subsequently digested with sequence level trypsin reagent (250 μl; 8 ng/μl trypsin in 25 mM NH_4_HCO_3_, pH 8.0). The trypsin concentration was based upon an estimate of approximately 3-6 μg protein per gel section and adjusted if necessary. The solution was then replaced by 25 mM NH_4_HCO_3 _to cover the gel pieces (100-200 μl) and incubated overnight at 37°C. If needed, additional ammonium bicarbonate buffer would be added to completely cover the gel pieces. After digestion, the supernatant of each tube was transferred to another Eppendorf tube. To extract residual peptides, the gel pieces were sonicated for 20 min at 37°C in a solution of 60% ACN/0.5% TFA. The extraction was repeated twice. The extracts combined with the primary supernatant were dried in a vacuum centrifuge for further separation by 2D-nanoLC/MS.

### Automated 2D LC-ESI-MS/MS separation and identification

All the extracted peptides of protein bands from SDS-PAGE maps were desalted using a 1.3 ml C18 solid phase extraction column (Sep-Pak^® ^Cartridge, Waters Corporation, Milford, USA). The dried peptides then were resuspended in loading buffer (5 mM Ammonium formate containing 5%ACN, pH 3.0), separated and analyzed by 2D strong cation-exchange (SCX)/plus reversed-phase (RP) nano-scale liquid chromatography/mass spectrometry. The experiments were performed on a Nano Aquity UPLC system (Waters Corporation, Milford, USA) connected to an LTQ-Orbitrap XL mass spectrometer (Thermo Electron Corporation, Bremen, Germany) equipped with an online nano-electrospray ion source (Michrom Bioresources, Auburn, USA).

A 180 μm × 2.4 cm SCX column (Waters Corporation, Milford, USA) packed with a 5 μm polysulfoethyla spartamide (PolyLC, Columbia, MD, USA) was used for the first dimension of separation. To recover hydrophobic peptides still retained on the SCX column after a conventional salt step gradient, an RP step gradient from 15% to 50% acetonitrile (ACN) was applied to the SCX column [55].

A 9 μl plug was injected each time to form the step gradients. The buffers used to form the gradients were prepared from a 1 M NH4FA stock solution, pH 3.20. The buffers used for the conventional salt gradient (5-500 mM) contained 5% ACN, while the buffers prepared to form the RP step gradient (10%-50% ACN) all contained 500 mM NH4FA. The organic solvent gradient was carried out following the salt gradient. The plugs were loaded onto the SCX column at a 4 μl/min flow rate for 4 min, with a steps as following:(a): 5 mM and 5%ACN, (b) 100 mM and 5%ACN,(c): 200 mM and5%ACN, (d): 250 mM and 5%ACN, (e): 300 mM and 5%ACN, (f): 400 mM and 5%ACN (g): 500 mM and 5% ACN, (h): 500 mM and 15% ACN, (i): 500 mM and 30% ACN,(j): 500 mM and 50% ACN. At last, 1 M Ammonium formate (NH4FA) was used to clean the SCX column twice. The eluted peptides were captured by a trap column (Waters Corporation, Milford, USA) while salts were diverted to waste. The trap column (2 cm × 180 μm) was packed with a 5 μm Symmetry^® ^C18 material (Waters Corporation, Milford, USA). The RP analytical column (20 cm × 75 μm) was packed with a 1.7 μm Bridged Ethyl Hybrid (BEH) C18 material (Waters Corporation, Milford, USA), and was used for the second dimension separation.

The peptides loaded on the RP analytical column were eluted with a three-step linear gradient. Starting from 5% B to 45% B in 40 min (A: water with 0.1% formic acid; B: ACN with 0.1% formic acid), increased to 80% B in 3 min, and then to 5% B in 2 min. The column was re-equilibrated at initial conditions for 15 min. The column flow rate was maintained at 300 nl/min and column temperature was maintained at 35°C. The 1.1 kV voltage electrospray versus the inlet of the mass spectrometer was used. LTQ-Orbitrap XL mass spectrometer was operated in the data-dependent mode to switch automatically between MS and MS/MS acquisition. Full-scan MS spectras with two microscans (m/z 300-1800) were acquired in the Orbitrap with a mass resolution of 60,000 at m/z 400, followed by ten sequential LTQ-MS/MS scans. Dynamic exclusion was used with two repeated counts, 10s repeated duration, and 60s exclusion duration. For MS/MS, precursor ions were activated using 35% normalized collision energy at the default activation q of 0.25.

### Peptide Sequence and Data Interpretation

Peptides ion eluted in salt gradient were identified by accurate molecular weight as a precursor ion and the product-ion spectrum was compared with the theoretical human protein index database after collision-induced dissociation (CID) of the precursor ion. All MS/MS spectrums were searched by SEQUEST (V28, revision 12, Thermo Electron Corporation) against the human International Protein Index (IPI) database. To reduce false positive identification results, a decoy database containing the reverse sequences was appended to the database. The searching parameters were set up as follows: two missed cleavages partial trypsin were considered, the variable modification was oxidation of methionine, the peptide mass tolerance was 10 ppm, and the fragment ion tolerance was 1 Da. Trans Proteomic Pipeline software (revision 4.0 Institute of Systems Biology, Seattle, WA) was then utilized to identify proteins based upon corresponding peptide sequences with more than 95% confidence. The peptides results were filtered by peptide prophet [56] with a *P*-value over 0.05 then a protein prophet [57] probability of 0.95 was used for the protein identification.

## Abbreviations

2D: two-dimensional; IHC: Immunohistochemistry; 2-DE: two-dimensional electrophoresis; MS: mass spectrometry; SDS-PAGE: Sodium dodecylsulfate polyacrylamide gel electrophoresis; MALDI-TOF: Matrix-assisted laser desorption/ionizationtime of flight mass spectrometry; ESI: Electrospray ionization; TBS: Tris-Buffered Saline; ACN: acetonitrile; SCX: strong cation-exchange; RP: reversed-phase; LTQ: linear trap quadrupole; LCM: laser capture microdissection; PRL: prolactin; nanoLC/MS: nano-scale liquid chromatography/mass spectrometry; PMF: Peptide Mass Fingerprinting; pI: isoelectric Point; MW: molecular weight

## Competing interests

The authors declare that they have no competing interests.

## **Authors' contributions**

YL performed LCM, data analysis and drafted the manuscript. JW provided the pituitary adenoma tissues, partly designed the experiment and participated in the writing of the manuscript. GY performed trypsin digestion and protein identification by mass spectrometry. RH contributed to the bioinformatics analysis. LC optimized SDS-PAGE. QP took part in experimental design, protein identification by MS and revision of the final manuscript. DZ assisted in immunohistochemistry and contributed to the final manuscript. JZ participated in experimental design, data analysis, coordination and revision of the final version of the manuscript. All authors read and approved the final manuscript.

## Supplementary Material

Additional file 1 **Prolactinoma proteome database. **Microsoft Excel spreadsheet (XLS format) containing the proteome database for prolactin cells isolated from prolactinomas by immuno-LCM. Click here for file
